# Contact tracing apps for the COVID-19 pandemic: a systematic literature review of challenges and future directions for neo-liberal societies

**DOI:** 10.1007/s13755-021-00147-7

**Published:** 2021-04-13

**Authors:** Alex Akinbi, Mark Forshaw, Victoria Blinkhorn

**Affiliations:** 1 School of Computer Science and Mathematics, Liverpool John Moores University, James Parsons Building, Liverpool, UK; 2 School of Psychology, Liverpool John Moores University, Tom Reilly Building, Liverpool, UK

**Keywords:** Contact tracing, COVID-19, Contact tracing apps, SARS-CoV-2

## Abstract

**Purpose:**

The COVID-19 pandemic has spread with increased fatalities around the world and has become an international public health crisis. Public health authorities in many countries have introduced contact tracing apps to track and trace infected persons as part of measures to contain the spread of the Severe Acute Respiratory Syndrome-Coronavirus 2. However, there are major concerns about its efficacy and privacy which affects mass acceptance amongst a population. This systematic literature review encompasses the current challenges facing this technology and recommendations to address such challenges in the fight against the COVID-19 pandemic in neo-liberal societies.

**Methods:**

The systematic literature review was conducted by searching databases of Google Scholar, Web of Science, PubMed, IEEE Xplore Digital Library, PsycInfo and ScienceDirect using the search terms (“*Contact Tracing*”* OR *“*Contact Tracing apps*”)* AND *(“*COVID-19*”* OR *“*Coronavirus*”) to identify relevant literature. The searches were run against the title, keywords, or abstract, depending on the search platforms. The searches were conducted between January 1, 2020, through 31st January 2021. Further inputs were also taken from preprints, published government and technical reports. We explore and discuss from the selected literature, the key challenges and issues that influence unwillingness to use these contact tracing apps in neo-liberal societies which include the plausibility of abuse of user privacy rights and lack of trust in the government and public health authorities by their citizens. Other challenges identified and discussed include ethical issues, security vulnerabilities, user behaviour and participation, and technical constraints.

**Results and conclusion:**

Finally, in the analysis of this systematic literature review, recommendations to address these challenges, future directions, and considerations in the use of digital contact tracing apps and related technologies to contain the spread of future pandemic outbreaks are presented. For policy makers in neo-liberal societies, this study provides an in-depth review of issues that must be addressed. We highlight recommendations to improve the willingness to use such digital technologies and could facilitate mass acceptance amongst users.

## Introduction

The novel COVID-19 disease caused by severe acute respiratory syndrome–coronavirus 2 (SARS-CoV-2) has rapidly spread with increased fatalities across the world leading to a worldwide pandemic. In the fall of 2020, new mutations and variants of this disease detected in the U.K and South Africa, B.1.1.7 and B.1.351 respectively, have since emerged and are circulating globally [1]. In response to the rapidly growing number of cases and the danger of overburdening health systems, many countries have resulted in lockdowns to slow the spread of the novel coronavirus [2]. Other strategies including mass testing and manual contact tracing (using humans or health professionals to collect data) have been deployed to contain the disease and to help ease lockdown restrictions. Like previous similar outbreaks such as Severe Acute Respiratory Syndrome (SARS) and Middle East Respiratory Syndrome (MERS), manual contact tracing and follow-up control measures such as quarantine and isolation were crucially important and successful during these outbreaks [3–6]. Although SARS and MERS were both considered as “fast-course” infectious diseases given their relatively short infectious period, the rate of infection and transmission of the SARS-Cov-2 virus is much faster and the death rate outweighs both SARS and MERS which on the scale makes manual contact tracing response labour-intensive, slow and imperfect [7]. One weakness of the manual contact tracing method is that it greatly relies on whether the confirmed cases can recollect who they met in the places where they have been or are willing to voluntarily disclose such information. Moreover, there is evidence that SARS-CoV-2 has a reproduction number of around 2–3 in the early stages of an outbreak and many infections can occur without symptoms which are higher than that for SARS (1.7–1.9) and MERS (< 1), suggesting that SARS-CoV-2 has a higher pandemic potential which requires a different approach [8–13].

Hence, new technology-based methods have been considered to fill the gap in the identification of contacts, especially if case detection is aggressive [14, 15]. Public health authorities and governments have responded by building digital contact tracing mobile apps like the ones initiated in Singapore, South Korea, and China to keep track of meetings between individuals which allow self-isolation instructions to be sent automatically to everyone when a newly diagnosed patient has interacted with them while infectious with the SARS-CoV-2 virus. Also, Apple and Google in a joint effort to assist announced a new technology for third-party apps on iOS and Android devices to support public health authorities around the world in developing digital contact tracing apps [16]. The concept relies on Bluetooth low-energy (BLE) beaconing technology to record when a phone has come into close-proximity with anyone else using the app to track and trace infections. Considering Google Android and Apple iOS jointly hold the highest market share of smartphone operating systems, it seems likely that their approach will be critical in how the majority of contact-tracing apps will function [17]. Although Apple and Google claim user privacy and security are at the core of the design, privacy concerns have also been raised noting that contact tracing apps can otherwise be repurposed to enable unwarranted discrimination and surveillance by governments on their citizens, or data harvesting by third parties [18].

This has created trust issues in the acceptance and participation of users to download and use such apps especially in neo-liberal societies where their use is not mandatory, hence defeating the real purpose of digital contact tracing. For contact tracing apps to be effective, roughly 50% to 70% of a population needs to use them [19] and scientific and epidemiological evidence suggest contact tracing apps have the potential to contribute to reducing the suffering caused by the pandemic and ease lockdown [20]. Strong support for the apps in online surveys carried out in neo-liberal countries like France, Germany, Italy, the UK, and the US, shows that the willingness to install contact tracing apps is very high [21]. However, this does not equate to people doing so. Recent studies show that these apps are not being used by enough of the population in countries including India, Norway, Singapore, and Iceland [22].

In a survey from the Washington Post and the University of Maryland, USA, respondents were split evenly (50–50%) on whether they will use a contact tracing app if one was made available. Of the 50% of respondents who said they will use it, only 17% said they would use it compared to 32% who said they will probably use it [23]. Despite the threshold set between 50 and 70% of the population’s use of the app to make it effective, recent statistics from countries where contact tracing apps are voluntary are not promising. At the time of writing, about one million people (20% of the population) have downloaded Singapore’s contact-tracing app ‘*TraceTogether*’ with 16% of the population currently being active users. Australia’s *COVIDSafe* app has been downloaded 6 million times based on data from the Google Play Store (4% of the population) and so far, just one person has been reported to have been identified using data from it. Similarly, the French contact tracing app ‘*StopCovid*’ with 1.9 million downloads from both the App Store and Play Store only managed to send 14 notifications since its release (at the time of this writing). Recent data from the app developers in the first 3 weeks since its release show from the 1.9 million downloads, 23,953 people deactivated the app while 460,000 people simply uninstalled the app without apparent reasons. The effectiveness of contact tracing apps to track and trace individuals infected with the novel SARS-Cov-2 virus has also been met with scepticism both by industry experts and academics in western democracies. Technical, privacy and security problems have hampered the apps and their impact on the COVID-19 pandemic remain uncertain [24, 25].

Considering the above, we conducted a systematic literature review (SLR) of the current challenges and application of contact tracing apps in the fight against the COVID-19 pandemic. The aims of this study and the main contributions are as follows:

- Provide an up to date review on the current challenges of contact tracing apps in the fight against COVID-19 in neo-liberal societies.
- Discuss recommendations to address these challenges.
- Explore future directions and considerations in the use of digital contact tracing technologies in the fight against future infectious disease outbreaks.

The remainder of this paper is organized as follows. In “Mandatory application of contact tracing apps in East Asia” section, we discuss the mandatory application of contact tracing apps for the COVID-19 pandemic in East Asia. In “Research Methodology” section, we describe the research methodology used for this study. Results from the systematic literature review are presented in “Results” section. In “Discussion of results” section, we discuss findings from the results of the study; highlight some of the key challenges; recommendations and identify future directions for contact tracing technology. Finally, in “Conclusion and future work” section, we conclude the paper and highlight potential areas for future research and investigation.

## Mandatory application of contact tracing apps in East Asia

Contact tracing apps can greatly support testing, tracing, isolating, and quarantine measures in the attempt to mitigate and slow the spread of the SARS-Cov-2 virus by speeding up processes of reporting and contact tracing through improved digital data flow, proximity tracing and geolocation tracking [26]. It could play a pivotal role given the ubiquity of internet-connected devices and increase the speed of surveillance of a large population of smartphone users in almost real-time to know where the infection hotspots are. As lockdown measures are gradually being lifted in many countries, contact tracing apps are central to control strategies during the de-escalation of social distancing [27]. It can also be crucial in flagging more infections especially in scenarios where manual contact tracing cannot. For example, a yet-to-be diagnosed person has the SARS-Cov-2 virus and takes a crowded bus to work; manual contact tracing is unlikely to identify everyone on the bus standing close to the infected person. Indeed, a study by Kucharski [28] found that combined testing and contact tracing strategies significantly reduced the reproductive number more than mass testing or self-isolation alone.

In East Asian countries such as China and especially Taiwan, contact tracing apps have been mandatory and proved effective alongside manual contact tracing methods in identifying new cases since the end of lockdown [25, 29, 30]. The apps generally work by assigning a colour code (green, yellow, or red) using an algorithmic assessment of the user’s travel history and health status. People who can show a green health code on their smartphones demonstrate they have not been in contact with a confirmed case of COVID-19 [31–33]. In South Korea, contact tracing apps such as the ‘*Corona 100*’ seems to be popular and has enabled public health officials to reduce the time needed to trace a patient’s movements from around 24 h to approximately 10 min and thus, helped the general public avoid infectious areas.

South Korea’s extensive contact tracing, testing, and isolation measures received overwhelming support from the population and have helped to reduce the virus’s spread [34]. Laws passed and data transparency during the MERS outbreak in 2015 allows the South Korean government to collect and publish public data including travel histories of confirmed patients. Hence, the privacy of the population has been given up to the government especially in response to public health safety. To ensure people under compulsory home quarantines do not stray from the confines of their apartments, the Hong Kong government required compulsory download of the ‘*StayHomeSafe*’ app and provided geofencing electronic tracker wristbands that alert authorities if they violated quarantines. These approaches have significantly contributed to the mass acceptance of contact tracing apps so far in the region.

There is little evidence to suggest that the use of these types of approaches adopted in East Asian countries might be successfully transferable to neo-liberal societies such as the USA, UK, France, Germany etc. with different political and cultural systems. Controversies about the legitimacy of these apps have largely focused on issues of privacy and surveillance as commentators emphasized the differences between populations in East Asia’s acceptance of state surveillance and a European scepticism towards this practice [35]. The barrier appears to be that many of these countries especially in Europe are very sensitive to privacy issues and privacy is protected by law like the General Data Protection Regulation (GDPR) [36, 37]. A study by Hernandez-Orallo [38] shows that for possible second waves of infection, contact tracing apps can be effective in controlling the SARS-Cov-2 virus, assuming that a percentage of the population will have gained immunity, or implemented in combination with some other lenient measures, such as social distancing. Moreover, for many of such countries that are resuming business operations and social activities, or where protests are happening and the number of social contacts increases, it will be worthwhile for them to invest in strategies to vastly improve the mass acceptance of contact-tracing apps to enable rapid response to a resurgence of the SARS-Cov-2 virus [39]. However, the apparent dilemma faced by neo-liberal governments is making a conscious choice between privacy and public health whilst showing the efficacy of such apps.

## Research methodology

The systematic literature review (SLR) was conducted by searching databases of Google Scholar, Web of Science, PubMed, IEEE Xplore Digital Library, PsycInfo and ScienceDirect using the search terms (“*Contact Tracing*”* OR *“*Contact Tracing apps*”) *AND* (“*COVID-19*”* OR *“*Coronavirus*”) to identify relevant literature. The search strings were run against the title, keywords, and abstract, depending on the search platforms. The searches were conducted between January 1, 2020, through January 31, 2021. Further inputs were also taken from relevant preprints, published government and technical reports. Previous studies [40–45] have used similar methods to conduct an SLR. To achieve the objectives of extensively reviewing the most relevant studies and answering the research questions. We conducted the SLR under the guidance published by Kitchenham and Charters [46]. According to Kitchenham and Charters, a Systematic Literature Review is “a form of secondary study that uses a well-defined methodology to identify, analyse and interpret all available evidence related to a specific research question in a way that is unbiased and repeatable” [46]. The SLR allows us to implement the three phases of planning the review, conducting the review, and reporting or documenting the review. Each phase of the SLR is outlined below:

Planning the review involves the following steps:

- Identification of the need for an extensive literature review
- Formulating the research questions (RQ1 to RQ3)
- Development of search strategy (this involves using search strings, sources selection, search processes and documenting search strategy).

Conducting the review involves the following steps:

- Selection of relevant publications (Inclusion and Exclusion Criteria)
- Evaluation and assessment of selected relevant publications
- Data extraction and synthesis-to address the research questions (RQ1 to RQ3).

Reporting the review (Documentation) involves the following steps:

- Presentation of results
- Discussion of findings based on the research questions (RQ1 to RQ3).

**Fig. 1:**
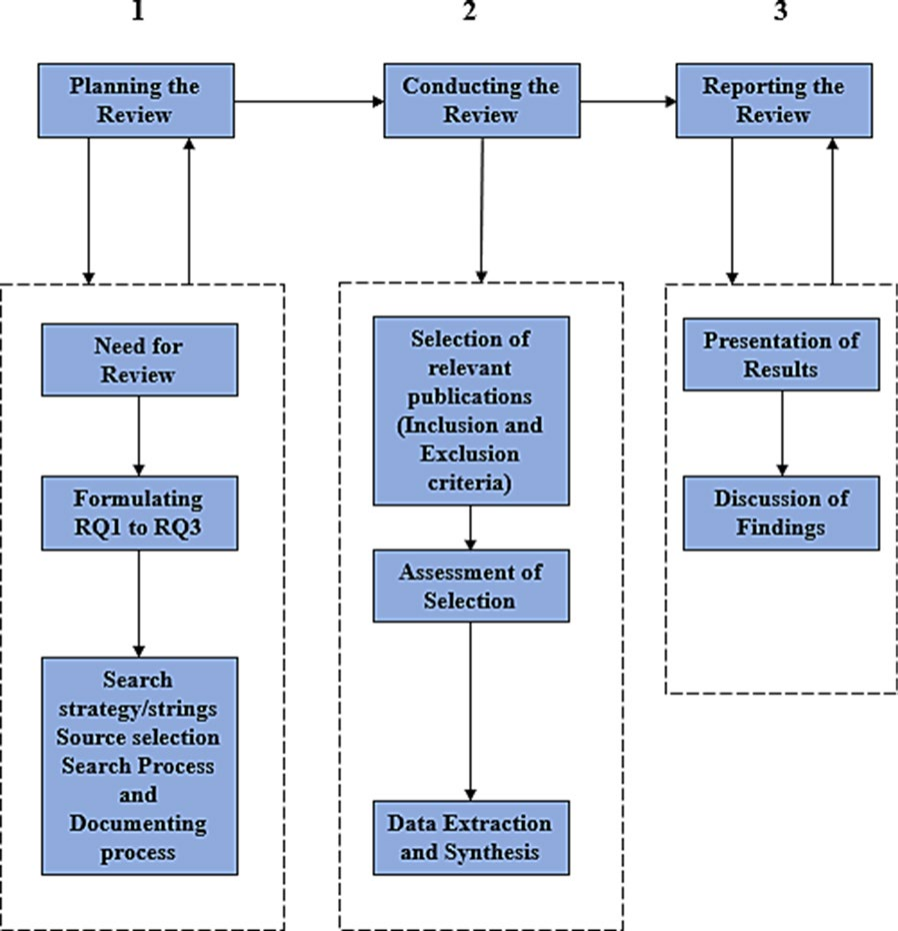
Phases of the Systematic Literature Review (SLR)

The phases of the SLR adopted to conduct this research is presented in Fig. 1.

### Planning the review

The purpose of this SLR is to analyse current and existing studies and their findings and to summarize the current challenges in the application of contact tracing apps in the fight against the COVID-19 pandemic in neo-liberal societies. To make sure our study is focused, we developed three research questions as follows.

1. What are the current challenges facing the efficacy and mass acceptance of contact tracing apps for COVID-19 in neo-liberal societies?
2. What recommendations can be implemented to address these challenges and improve mass acceptance?
3. What are the future directions and considerations in the use of digital contact tracing technologies in the fight against future pandemic outbreaks?

The study complements existing studies by conducting an SLR to identify the pressing challenges related to the adoption of contact tracing apps in the fight against COVID-19, a global pandemic that has claimed a significant number of human lives and caused major disruptions to business, led to social isolation and modified human relations in neo-liberal societies up to January 2021. It provides an up to date study and the current state of contact tracing apps in containing the spread of this deadly infectious disease. The study aims to present governments and policymakers with the challenges they face and discuss strategies they need to consider if the population are to adopt the use of contact tracing apps now and similar contact tracing technologies in future pandemic outbreaks.

### Conducting the review

These searched were conducted on the 31st of January 2021. A total of 18,566 results were returned from the initial searches carried out using the search strings and keywords on the online digital databases. These results included a combination of peer-reviewed publications, preprints, and reports from credible sources. The results from these searches were filtered through the inclusion/exclusion criteria to remove irrelevant and duplicate publications. Moreover, we implemented forward and backward snowballing iterations [47] to ensure that all the selected publications were relevant and met the inclusion criteria. These inclusion criteria were:

- Selected publications must be relevant to contact tracing technology and its application in the fight against the COVID-19 pandemic.
- Publications must be related to the research questions (RQ1 to RQ3).
- Publications must be written in English.

The exclusion criteria, on the other hand, were:

- Publications not relevant to contact tracing technology and its application in the fight against the COVID-19 pandemic.
- Duplication of published sources, news articles and literature not written in English.

Since an SLR is about conducting a comprehensive search of all relevant sources related to the research topic, further checks using a quality assessment was applied for a more rigorous result. A detailed assessment of the selected publications was done based on the checklist as set by Kitchenham and Charters [46] to determine their relevance and suitability for addition to the SLR. The quality assessment was based on the primary goal of the selected publication, context, and relevance to the stated research questions RQ1 to RQ3. We selected 5 publications at random and used the following quality assessment check for the final selection of publications. The publication must meet at least one or more following.

1. **Contact Tracing Apps.** The publication must include discussion and application of contact tracing apps in the fight against COVID-19.
2. **Context.** The content of the publication consists of relevant details which explain the objectives and findings.
3. **Challenges of Contact Tracing Apps**. The publication consists of some significant details on the challenges of digital contact tracing apps in the fight against COVID-19 to address RQ1.
4. **Recommendations.** The publication includes some recommendations to address challenges of digital contact tracing or the use of contact tracing apps in the fight against COVID-19 to address RQ2.
5. **Future Considerations.** The publication discusses future considerations in the implication or use of digital contact tracing technology in the fight against future pandemics to address RQ3.

**Fig. 2:**
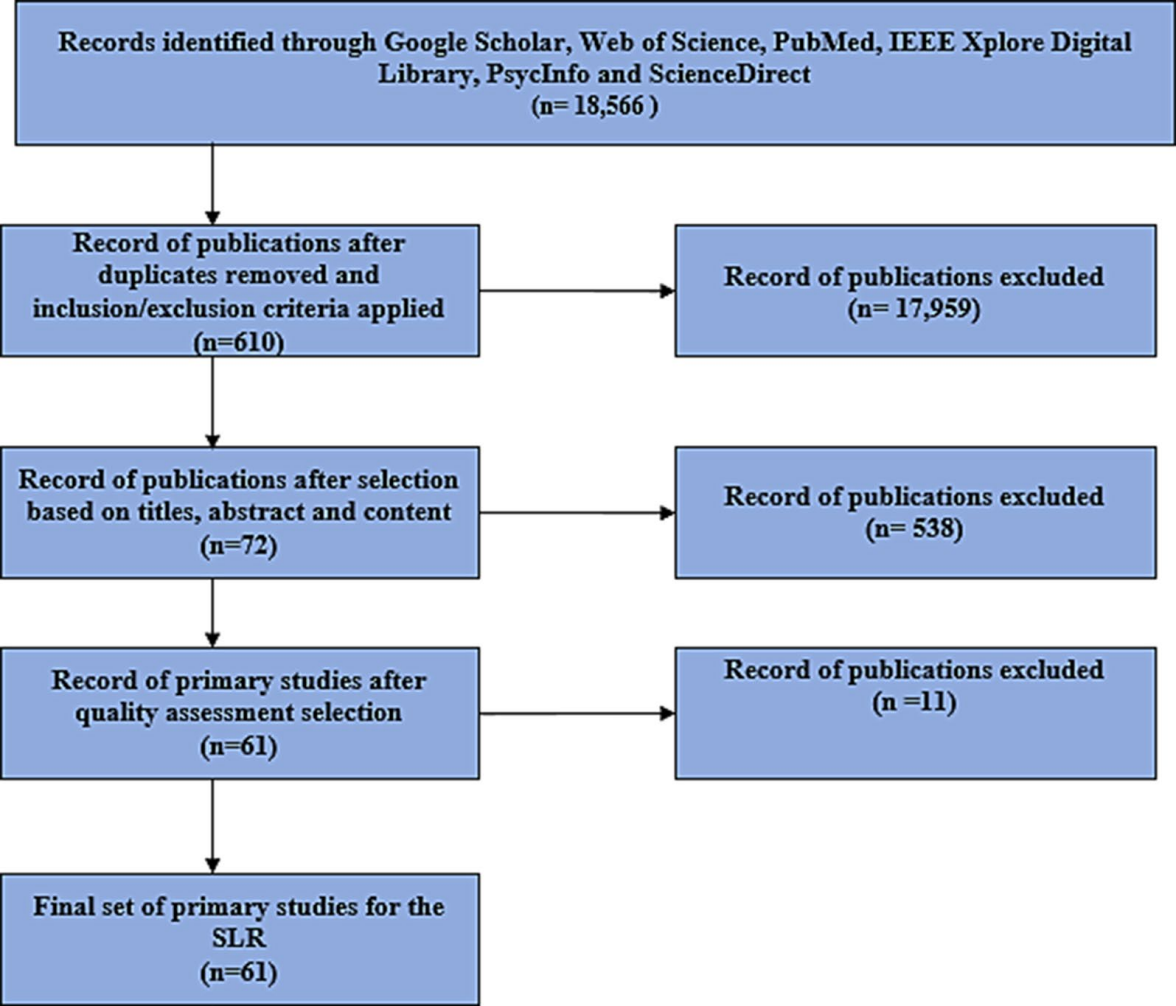
Selection of primary studies for the SLR

With this process, 17,959 publications were excluded from the initial search results, bringing the total number down to 610 publications. Following that, the exclusion criteria based on titles, abstract and content was implemented; and as a result, 538 publications were also excluded altogether, bringing the number down to 72 relevant publications. Finally, 61 publications were identified as the final set of primary studies for this SLR after the quality assessment selection. Figure 2 shows the number of publications selected and excluded at each stage of the process. The data extracted from the collection of relevant publications were used to provide answers to the research questions RQ1 to RQ3 in line with the objectives of this study. The data extraction and synthesis for the final publications are described and categorised based on the following categories:

- **Context Data:** Details about the aim and purpose of the study.
- **Qualitative data:** Findings and conclusions from the relevant study.
- **Quantitative data:** Results from extracted data, discussions, and findings from the relevant study.

## Results

**Fig. 3:**
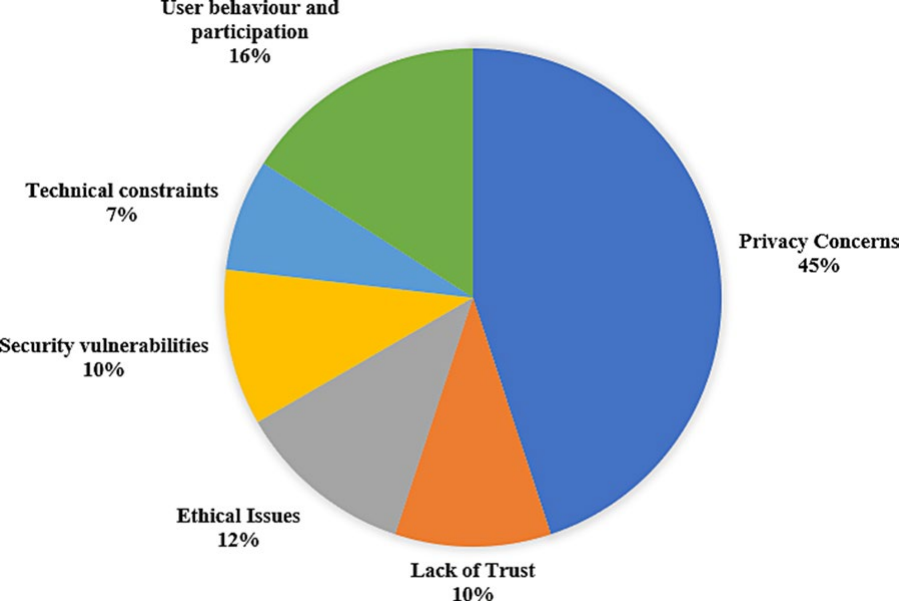
Chart of themes of primary studies

Each publication from the final set of 61 primary studies was read in full after they passed the quality assessment and relevant context data, qualitative and qualitative data was extracted. All primary studies are centred on the research questions RQ1 to RQ3 and a theme in relation to the challenges, recommendations and future directions of contact tracing apps and related technology in the fight against COVID-19 and future pandemic outbreaks. Figure 3 shows the percentages of different themes of the 61 primary studies, after the quality assessment selection and were included in the analysis and discussion of results. Most research outputs considered in this SLR are published in academic journals. Also, there were few outputs published as preprints, government, and technical reports.

The themes identified in the primary studies show that almost half (45%) of all studies on contact tracing apps for the COVID-19 pandemic are concerned with user privacy. User behaviour and participation is the second most popular theme amongst the primary studies, with a percentage of 16%. This is followed by ethical issues surrounding the use of contact tracing apps in 12% of the primary studies. Lack of trust in the government especially for neo-liberal societies accounts for 10% of the primary studies. The studies focused on the plausibility of contact tracing apps being used for government surveillance as one of the reasons behind the unwillingness of the population to use them. Security vulnerabilities are the jointly third commonest theme, with a proportion of 10%. The last common theme of the primary studies is technical constraints that affect the adoption of contact tracing apps, which accounts for 7%.

## Discussion of results

The decision to develop and deploy contact tracing apps for tracking and tracing the spread of the COVID-19 pandemic continues to raise data privacy concerns and a balance between user data privacy and societal benefit has been considered [48]. This coupled with its effectiveness, ethical considerations, security risks, and technical issues has been highlighted as major challenges affecting mass acceptance amongst the population in neo-liberal societies. In this section, we present findings from the selected primary studies that highlight the major challenges affecting contact tracing apps in the fight against COVID-19, recommendations to address these challenges and future directions in the use of digital contact tracing technology to fight future pandemics.

### RQ1: what are the current challenges of contact tracing apps in neo-liberal societies?

It is important to state that this systematic literature review focuses on the challenges of contact tracing apps that influence their wide acceptance and adoption, especially in neo-liberal societies. However, it is imperative to also note that some of the discussion in our findings could apply to other demographics and digital applications.

- *User data privacy concerns*—Parker et al. [20] highlights that justification for privacy infringements of users is hypothetically justifiable in cases where contact tracing apps have the potential to contribute to the saving of many lives and reduce enormous suffering caused by a blanket population lockdown. Hence, people should be prepared to trade-off privacy encroaching contact tracing apps for civil liberty and see it as a public duty to save lives as lockdown is being eased. However, an app encroaching on people’s privacy while providing little contribution, compared to other measures in tackling the spread of COVID-19 would be ethically dubious, especially in neo-liberal societies [49, 50]. Privacy concerns related to user data has been of one the significant issue affecting the acceptance and willingness to use contact tracing apps as shown in primary studies [37, 51–60]. Questions such as how user data will be anonymized, where the data will be stored, who has access to the data, how it will be shared, used and destroyed when the pandemic is over have been subjects of huge debate. These are the main reasons that influence unwillingness to use these contact tracing apps.
- *Lack of Trust—*The lack of trust in government and their motive appears to be a key factor that creates a negative effect on people’s decisions to install a contact tracing app on their phones especially in neo-liberal societies where the use is not mandatory and success depends on the establishment of sustained and sound public trust and confidence as shown in primary studies [21, 61, 62]. Especially in the USA in the post 9–11 era, lack of the public’s trust in governments has been impacted since Edward Snowden’s revelations on US government global surveillance program.
  Apart from surveillance concerns associated with central authorities’ access to user data, concerns associated with access to user data by third parties have also been raised [60]. These include any individual with whom a user has exchanged tokens in the contact tracing app based on some notion of physical proximity, big data analysis companies, or malicious actors where the contact tracing app’s system is naïve or vulnerable to information leakage [53]. In May 2020, the official COVID-19 contact tracing app for the state of North Dakota, USA, was found to send user location data and the unique user identifier to Foursquare and other data to Google including a bug-tracking company with the users’ consent [63].
  A scientific Data Protection Impact Assessment (DPIA) of contact tracing app designs (including centralized and decentralized models) conducted in the primary study [64] found that none of the proposed designs ensures proper anonymization, and that informed consent would not be a legitimate legal ground for the processing, that data subjects’ rights are not sufficiently safeguarded and that no design provides for sufficient purpose-binding. Similar findings in the application of the European Data Protection Board (EDPB) guidelines in the assessment of three contact tracing apps (*Stopp Corona*, *NHS COVID-19* and *TraceTogether*), showed varying compliance with the guidelines criteria [54]. The issues highlighted show that maintaining the balance between trust in government institutions and public health is a huge challenge in the adoption of contact tracing apps in the fight against COVID-19.
- *Ethical issues—*In many countries, residents have been living under lockdown with their civil liberties heavily curtailed. Many businesses have been forced to stop operations forcing millions out of work. According to the International Monetary Fund’s (IMF) April World Economic Outlook, the global growth in 2020 is expected to fall to -3% making it the worst recession since the Great Depression [65]. The United Kingdom’s economic GDP is projected to fall by 11.5% while the USA, China, Germany, and France GDPs are predicted to fall by 6.6%, 2.6%, 6.6%, and 11.4% respectively [66]. As countries slowly lift restrictions and business open to enable quick economic recovery, digital contact tracing itself can contribute to general fairness risk associated with discriminate mitigation measures [67]. For example, disadvantaged workers are less likely to be able to work from home, engaging in work means that they are at higher risks of becoming infected and are more likely to form social ties with others in similarly precarious arrangements. Therefore, they may be forced to quarantine simply because they have been in close proximity with others in the same social group although they may not be at high risk of infection. The lack of smartphones and internet access, as well as the share of informal employment all come together to disproportionately impact the low-income communities which continue to drive the health divide rooted in social status and economic differences even further [68, 69].
  Primary studies [64, 70–72], discuss the risk of data collected through contact tracing apps by public health authorities and governments can be used not just for epidemiological studies and surveillance but also for behavioural profiling of a population. The behavioural profiles if correlated with other demographic and socio-economic data may motivate selective policies in which a population that has been measured as being on average more willing to take risks are treated differently by future restrictions than other groups whose compliance is supposedly higher. This raises serious ethical issues as data can be used to discriminate against a population or geographic locations where cases of COVID-19 are higher. For example, behavioural scoring can be used to determine access to medical resources, funding, treatment, etc. It was discovered that Bahrain’s ‘*BeAware Bahrain*’ app has been sharing data with a national television show called Are You at Home? which offered prizes to those who stayed at home during Ramadan [73]. In South Korea, contact-tracing laws permit the government to determine the immigration status of infected individuals. Sinha and Paterson [17] highlight that if such laws exist in the U.S., undocumented communities may not seek healthcare and that over time the same technologies and laws could be used to track undocumented migrants.
- *Security vulnerabilities—*Security flaws in the design and implementation of contact tracing apps have the potential to put sensitive personal details of users at risk. A recent investigation by Amnesty Security Lab discovered a significant weakness in the configuration of Qatar’s mandatory *EHTERAZ* contact tracing app [74]. The vulnerability could allow hackers access to highly sensitive personal information, including the name, national ID, health status, and location data of more than one million users. Similar vulnerabilities have been disclosed in India’s *Aarogya Setu* and Pakistan’s *Covid-19 Gov PK* apps. These vulnerabilities were from apps that have been tested by security researchers so far. Other proposed contact tracing apps may have similar or different security flaws that make them susceptible to attacks and data leaks.
  The primary study [75] described possible attacks on Bluetooth technology used by contact tracing apps. Recent Bluetooth vulnerabilities include BlueFrag (CVE-2020-0022 which affected Android devices running Android 8.0 to 9.0) and Bluetooth BIAS Attack (affected multiple Android and iOS devices) [76] were disclosed in February and May 2020 respectively and required patching. However, many Android devices did not receive this update as more than one billion Android devices around the world are no longer supported by security updates, leaving them potentially vulnerable to attacks. According to Google’s data from 2019, around 40% of Android active users worldwide are on version 6.0 or earlier and no longer receive security updates [77]. A successful Bluetooth BIAS attack would allow a malicious actor to impersonate a device from a previous secure Bluetooth connection pairing between two devices. This can be leveraged to conduct social engineering attacks or take control of the vulnerable device as contact tracing apps always require Bluetooth to be enabled to function. Other possible attacks and vulnerabilities susceptible to contact tracing apps are described in primary studies [78–81].
- *Technical constraints—*The development and roll-out of contact tracing apps revolve around several assumptions that raise questions about its efficacy to advance public health in the fight against COVID-19. These assumptions are that a large percentage of the population have access to compatible smartphones and an internet connection, the application’s design, that Bluetooth signals are accurate, and that people will choose to install and use the apps [61, 68, 82].
  According to Statista, the current number of smartphone users in the world today is 3.5 billion, and this means 44.98% of the world’s population owns a smartphone [83]. From this percentage, smartphones running the Android operating system held an 87% share of the global market in 2019 compared to the mobile operating system developed by Apple (iOS), which had a 13% share of the market [84], thus making Android the most popular mobile operating system used across the world. However, more than one billion Android devices are 2 years or more out of date and do not receive updates from device manufacturers and carriers. This means many Android devices may not benefit from updates to the new COVID-19 contact tracing system Google has built in collaboration with Apple. For example, the *TraceTogether* app requires Android 5.1 or higher, while the *CovidSafe* app works on Android 6.0 or higher. On iOS devices, both *TraceTogether* and *CovidSafe* require iOS version 10 or higher [78]. Currently, most back-end systems and contact tracing apps are poorly inter-connected since they are developed by different government agencies, health authorities, and organizations [85].
  Access to mobile internet across the world is not evenly distributed. According to the latest report from the GSMA Mobile Economy [86], smartphone subscriber penetration is considerably low in Sub-Saharan Africa (45%) compared to other regions like Europe (86%), North America (83%), Greater China (82%) and Asia Pacific (60%). This disparity is closely related to the GDP per capita of countries where citizens from poorer countries are less likely to own a smartphone. Even in regions like Europe, not all households have access to mobile internet and smartphones. In the UK, figures released by the Office for National Statistics (ONS), show that a third of households still do not have access to mobile broadband [87]. The latest figures in 2020 show that 30% of UK senior citizens aged 55 years and above do not own or have access to a smartphone and an estimated 21% of young adults aged 18 years and above do not have a smartphone [88]. This digital divide and inequality mean that health care development in the form of contact tracing apps and related technologies rapidly does not become available to many people within the population and has the unintended but inevitable consequence of fuelling health inequality [89].
  Bluetooth signals suffer from accuracy problems and will require developers to fine-tune how signals will be transmitted by lowering the transmission power to prevent such long-distance reception. Primary study [90] highlighted different Bluetooth versions and smartphone chipset implementations that can result in different operational and information security aspects of its use for contact tracing. Signal strength can vary significantly depending on the relative orientation of smartphones, on absorption by the human body, reflection, or absorption of radio signals in buildings and on trains. According to the inventors of Bluetooth, Jaap Haartsen, and Sven Mattisson, the signal’s path loss will vary significantly depending on extenuating conditions (free space or obscured) [91]. Duration, proximity, and direction of signal strength between two devices would also need to be measured to deal with problems associated with false positives. Especially in scenarios where the contact tracing app detects a non-valid exposure or false negative, where the app fails to detect a valid exposure because the distance was miscalculated, or even because of other external factors and extenuating circumstances. The inevitable danger of non-valid exposure measurements is linked to the risk that users are wrongly isolated, potentially several times in succession, with considerable economic and social consequences to those affected [15, 64]. The SARS-CoV-2 virus could spread by touching an object or surface with a virus present from an infected person, and then touching the mouth, nose, or eyes. Hence in such cases, a contact tracing app will not be able to detect this and may give people a false sense of security.
- *User behaviour and participation—*Most citizens over the age of 65 are not tech-savvy compared to the younger generation. Using the latest data on smartphone usage by age, sourced from the UK’s communications regulator OFCOM, there is a digital divide between adults of certain age groups and their attitudes towards the use of smartphones with young adults more likely to use contact tracing apps compared to senior citizens in the UK [89]. This intergenerational divide is supported by the study of participants in Singapore demonstrated in a primary study [92]. The success of any voluntarily installed app, for whatever purpose, is dependent upon user buy-in. There are several dimensions to that judgment of acceptability of technology, including its benefits and disbenefits, the moral or social imperatives, the perceived efficacy of the app, and the behaviour of significant others in the lives of the potential user. Psychological research into the adoption of technologies focuses on these issues, where wetware meets hardware and software.
  Primary studies [93–95], highlights adoption is determined by individual risks, cultural difference and social preferences, not by the wider health benefits to society. However, primary study [96], highlighted that a perceived realistic threat to life or health during the pandemic is a more important predictor of acceptance of contact tracing technologies in Poland. The study also found women were more willing to accept their use than men. Primary study [97] concluded that it is difficult for policymakers to design one app that fits all individuals in a society. Especially when the propensity to accept such non-mandatory apps varies between critics, advocates, and undecided individuals amongst the neo-liberal population. Tracking or screening technologies can work when the public understands the value of them for their health and wellbeing, and both parties are signed up for that greater purpose [98]. Under such conditions, surveillance concerns are minimized. This is confirmed by primary study [95] in a qualitative, focus-group study of participants in the U.K. Primary study [99] report a study of intentions to use a contact-tracing app in Australia, with the conclusion that uptake of the software can be increased if the security concerns are addressed, but that message framing did not make a significant difference to intention when autonomy-controlling and supportive messages were compared. Supportive messages involve choice and freedom, whereas autonomy-controlling messages use model verbs such as “should” and “must”.

Discussion from the primary studies shows an overwhelming consensus that privacy concerns is the most significant reason for the lack of acceptance and use of contact tracing apps amongst the population in neo-liberal societies. This issue is largely influenced by the cultural differences in these societies, lack of evidence of wider health benefits of these apps and growing concerns that government agencies could use data collected for digital mass surveillance.

### RQ2: what recommendations can be implemented to address these challenges and improve mass acceptance?

Based on the current challenges of contact tracing apps in the fight against COVID-19 in neo-liberal societies identified in RQ1, we discuss recommendations that can be applied to remedy these issues and can influence its wide adoption and acceptance:

- *Addressing user data privacy concerns—*Maintaining a balance especially between user privacy and societal benefit is a huge challenge if digital contact tracing using mobile apps is to succeed in the fight against COVID-19. Also, influencing user behaviour in the participation, and dealing with technical constraints associated with the underlying technology is essential if contact tracing apps are to succeed now and in respect of dealing with future pandemics. In this section, we discuss recommendations in addressing some of these issues and future considerations in the development and implementation of digital contact tracing. Data collected from such apps should only be used to support public health measures, the source code should be made public and subjected to public analysis and finally, its use must be voluntary, used with the explicit consent of the user and the systems must be designed to be able to be switched off, and all data deleted when the current pandemic is over [100]. There are suggestions that a non-partisan independent committee with representatives from legal, health, and privacy experts should be established to oversee the development of contact tracing apps, its information ecosystem, and data governance. Only anonymized aggregated data should be shared with public health authorities and any personal identifiable information must be deleted once the pandemic is over [60, 101].
- *Addressing security vulnerabilities—*On issues associated with Bluetooth, it is recommended that the Bluetooth Low Energy (BLE) signal should be regulated in a standardized manner when operating a contact tracing app so that the effective range of the protocol is reduced [90]. Legislation should be put in place which mandates smartphone manufacturers and carriers to provide critical system updates especially for Android devices vulnerable to critical Bluetooth vulnerabilities such as BlueFrag and BIAS attack. Mitigations to address security vulnerabilities promptly, application code review and secure software development must be considered to minimize risk to user data [60].
- *Addressing technical constraints—*Proximity accuracy issues with Bluetooth technology has been addressed in the recent update of Bluetooth Low Energy (BLE). However, there is still room for improvement in the development of new protocols and refined calibration of BLE signal strength that can enhance this technology [59, 78, 79].
- *Improving user behaviour and participation—*Currently, there a few studies about the psychological factors that would influence the adoption of an app for contact tracing at present for the ongoing outbreak of COVID-19. Findings from the primary studies [93, 95], suggests that people would respond best to messages which alleviate their security concerns, emphasize personal autonomy, and where the societal benefits are clearly articulated. It is also important that policy makers study their demographic to understand user perception. Designing a contact tracing app that targets most of the population and addresses their concerns (privacy and usability) can increase mass acceptance. Moreover, to realize their intended societal benefits, contact tracing apps require mass acceptance [97].

Our findings reveal that most of the primary studies consider showcasing the societal benefits of contact tracing apps, addressing privacy concerns, technical constraints and security issues that can influence the mass acceptance and use of contact tracing apps in neo-liberal societies.

### RQ3: what are the future directions and considerations in the use of digital contact tracing technologies in the fight against future pandemic outbreaks?

Since there are several underlying challenges of digital contact tracing apps in the light of their relevance in the fight against the COVID-19 pandemic, many of the issues are inherited from mobile applications as well. Our study shows that user privacy concerns are the most pressing challenge identified by most of the studies in their analysis of the implications of contact tracing apps, especially for neo-liberal societies. One of the positives of these apps is their ability to track and trace the spread of the infection in real-time whilst complementing other manual contact tracing methods. Despite these, for contact tracing apps to be effective, a large number of the population would need to install and use these apps. Based on the outcome of this SLR and our findings, we present the following future considerations and directions for contact tracing apps and related technologies in the fight against COVID-19 and future pandemic outbreaks that are worth investigating and implementing to encourage willingness and mass adoption by the wider population:

- *Adopting less-invasive and privacy-preserving technologies—*For future considerations, the use of less-invasive technologies such as Artificial Intelligence (AI) and Machine Learning (ML) has been proposed to help analyse the level of infection by the SARS-CoV-2 virus by identifying hotspots, tracing, and monitoring infected persons as described in primary studies [42, 78, 102–109]. Other methods described in the primary study [110, 111], propose the use of thermal-based imaging using the Internet of Medical Things (IoMT) and other Internet of Things (IoT) devices [112, 113] to trace and track positive cases and help control the spread of COVID-19 infection and future infectious disease outbreaks. The use of a privacy-preserving contact tracing scheme in blockchain-based medical applications has also been proposed [114, 115].
- *Transparency—*To encourage the willingness to adopt and use contact tracing apps, policymakers, developers, governments, and public health authorities in neo-liberal societies must adopt a feedback mechanism during the phases of deployment to create public confidence, trust and participation. Citizens deserve clarity on the purpose of data collection, types of data collected, who has access to such data, the modalities, extent and timeline for data deletion [35, 58, 59, 71, 78, 94, 116].
- *Influencing human behaviour—*It is important to study human behaviour when designing and developing contact tracing apps and related digital technologies before deploying and integrating them amongst the population. This includes studying a significant amount of theories and models such as the technology acceptance model, innovation diffusion theory, the theory of reasoned action, health belief models and theory of planned behaviour, social cognitive theory, and motivation theory can be used to explore the acceptance and use of future contact tracing technologies [85, 94].
- *Ethical considerations—*For government, technology developers, decision-makers and public health authorities, there is the need to translate the ethical–legal considerations into actionable safeguards that can unlock the promise of contact tracing apps and related digital technologies while avoiding harm and managing risks in the fight against future pandemics [71].

Based on the results of this survey and our observations, digital technologies can be used to support manual contact tracing and tracking methods in the fight against COVID-19 and future pandemic outbreaks. However, neo-liberal governments and public health authorities should consider the use of alternative technologies that do not invade user privacy. They also need to be transparent with the public on how any data collected will be used. Strategies and incentives to influence user participation should also be considered well in advance to encourage mass acceptance amongst the wider population.

## Conclusion and future work

The impact of the COVID-19 pandemic represents an unprecedented challenge to public health authorities and respective governments across the world. This has brought severe pressure on health services and introduced radical changes to the way of life for both individuals and organizations. In a way to stop the infection of the SARS-CoV-2 virus from spreading, public health authorities have considered and introduced robust contact tracing systems which include the use of digital contact tracing apps. In this paper, we discussed the mandatory application of contact tracing apps in East Asia in containing the spread of the SARS-CoV-2 virus and the challenges faced by neo-liberal societies in their use to fight against the COVID-19 pandemic. Although contact tracing apps are a promising technology for rapid tracing and tracking of infected persons, they can support manual contact tracing and tracking methods in the control of the SARS-CoV-2 virus. However, many people have an intrinsic mistrust of the government especially in neo-liberal societies and are concerned that the use of contact tracing apps could be the beginning of more pervasive government surveillance. Also, since these apps are not mandatory, it is difficult to predict mass acceptance and participation. If contact tracing apps are to succeed, it is important governments and policymakers gain the trust of their citizens and show adequate transparency in how user data is collected and used. Its efficacy and how these challenges are currently addressed in the fight against this novel disease will determine the role of digital contact tracing technologies in future pandemic outbreaks and what lessons can be learned from identified inadequacies.

Future potential research agenda concerning the impact and effectiveness of contact tracing apps and related technologies in neo-liberal societies needs to the considered. Studies that evaluate the effectiveness of the recommendations implemented by various policymakers, governments, and public health authorities to verify whether they influence the willingness and mass acceptance of this technology also needs to be carried out.
